# NGenomeSyn: an easy-to-use and flexible tool for publication-ready visualization of syntenic relationships across multiple genomes

**DOI:** 10.1093/bioinformatics/btad121

**Published:** 2023-03-08

**Authors:** Weiming He, Jian Yang, Yi Jing, Lian Xu, Kang Yu, Xiaodong Fang

**Affiliations:** BGI-Sanya, BGI-Shenzhen, Sanya 572025, China; BGI-Shenzhen, Shenzhen 518103, China; Key Laboratory of Neuroregeneration, Ministry of Education and Jiangsu Province, Co-Innovation Center of Neuroregeneration, Nantong University, Nantong, Jiangsu 226001, China; BGI-Sanya, BGI-Shenzhen, Sanya 572025, China; Key Laboratory of Neuroregeneration, Ministry of Education and Jiangsu Province, Co-Innovation Center of Neuroregeneration, Nantong University, Nantong, Jiangsu 226001, China; BGI-Shenzhen, Shenzhen 518103, China; BGI-Sanya, BGI-Shenzhen, Sanya 572025, China; BGI-Shenzhen, Shenzhen 518103, China

## Abstract

**Summary:**

Large-scale comparative genomic studies have provided important insights into species evolution and diversity, but also lead to a great challenge to visualize. Quick catching or presenting key information hidden in the vast amount of genomic data and relationships among multiple genomes requires an efficient visualization tool. However, current tools for such visualization remain inflexible in layout and/or require advanced computation skills, especially for visualization of genome-based synteny. Here, we developed an easy-to-use and flexible layout tool, NGenomeSyn [multiple (N) Genome Synteny], for publication-ready visualization of syntenic relationships of the whole genome or local region and genomic features (e.g. repeats, structural variations, genes) across multiple genomes with a high customization. NGenomeSyn provides an easy way for its users to visualize a large amount of data with a rich layout by simply adjusting options for moving, scaling, and rotation of target genomes. Moreover, NGenomeSyn could be applied on the visualization of relationships on non-genomic data with similar input formats.

**Availability and implementation:**

*NGenomeSyn* is freely available at GitHub (https://github.com/hewm2008/NGenomeSyn) and Zenodo (https://doi.org/10.5281/zenodo.7645148).

## 1 Introduction

With advances of the next-generation and long-read sequencing technologies, large-scale genome and pan-genome projects are largely increased, e.g. the Bird 10K Project (Zhang *et al.*, 2015) and rice pan-genomes (Qin *et al.*, 2021). Synteny analysis in comparative genomics is vital in understanding molecular-level similarities and differences in genome evolution and species diversity. Popular tools for the identification of syntenic blocks by comparison of two or more genomes based on whole-genome alignment or conserved gene anchors include SyRI (Goel *et al.*, 2019), MCScanX (Wang *et al.*, 2012), and GENESPACE (Lovell *et al.*, 2022). Circos is a popular circular visualization of genomic elements and relationships in comparative genomics, including synteny of two genomes (Krzywinski *et al.*, 2009). But rectangular chromosomes are more frequent used in visualization of synteny across multiple genomes (Hu *et al.*, 2019; Zheng *et al.*, 2022). Flexible in layout of visualization of gene-based synteny analysis on multiple genomes has been implemented in Jcvi (Tang *et al.*, 2015) and SynVisio (Bandi *et al.*, 2022). However, genome-based strategy is frequently used to detect structural variation among homologous genomes and independent of gene annotation. Though several visualization applications for genome-based synteny analysis have been recently developed, such as GenomeSyn (ZHou *et al.*, 2022) and plotsr (Goel and Schneeberger, 2022), tools for more flexible layouts and customizable visualization of syntenic relationships and structural variation (identification using gene- or genome-based methods) across multiple genomes, especially for pan-genomes which consist multiple *de novo* assembles of different accessions, are still limited. Here, we developed NGenomeSyn, an easy-to-use tool for visualization of syntenic relationship on chromosome-level or zooming in on regions of interest across multiple genomes with flexible layouts and high customization but not limited on genomic data. The NGenomeSyn is executable from the command line with the Perl language which makes tasks performed in a batch mode and reproducible.

## 2 Usage

NGenomeSyn requires only two options in the command line: one for a configuration file and the other for an output, and generates figures in SVG (Scalable Vector Graphics) and PNG formats. Required files of sequence length and links between two genomes, and optional files of highlighted special regions (e.g. Single-Nucleotide Polymorphism; Transposable element, TE; genes) should be provided in the configuration file. NGenomeSyn adopts a simple input format for sequence length and highlight of special regions with at least three ordered columns (sequence ID, start and end), and subsequent optional unordered fields could be set for attributes [e.g. feature type (CDS, UTR), color for stroke and fill; e.g. “fill=green”] for a special sequence or genomic region. For link files, the first six columns should be given two intervals of syntenic blocks [sequence (seq)A, startA, endA, seqB, startB, endB] between two genomes (e.g. genomes A and B) and subsequent optional unordered fields could be set attributes (e.g. color for stroke and fill) for highlight of a special syntenic block. For the convenience, we provided a pipeline for users to easily prepare input files (sequence length and links) for NGenomeSyn from two genomes (fasta format) using either Minimap2 (Li, 2018) or MUMmer (Delcher *et al.*, 2002) performing whole-genome alignment and then optionally call synteny and structural rearrangement using SyRI, or directly convert output from Minimap2, MUMmer or MCScanX.

**Figure 1:**
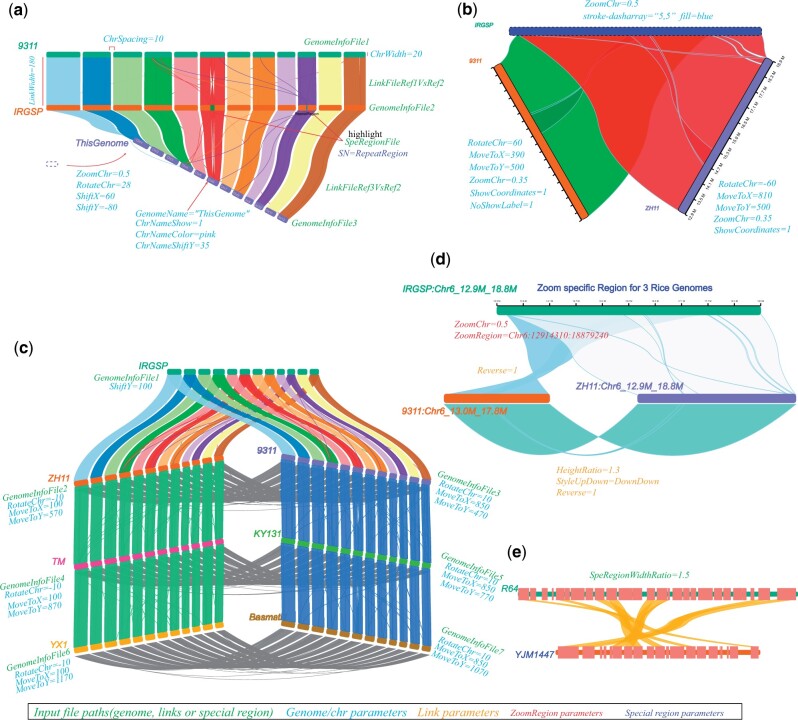
Customizing plots generated by NGenomeSyn. (a) General parameters for input files and attributes for genomes, links, and genomic special regions. (b) Triangular layout of three genomes. (c) A complex layout of seven genomes. (d) Syntenic regions of zooming in on given regions in three genomes. (e) Customizing height and color for special feature (e.g. CDS) in given regions of two yeast genomes. Colorful italic texts indicate parameters for input, genomes, special genomic regions, and links. Rice datasets were used to generate figures in panel a–d. Yeast datasets were used to generate figure in panel e.

In addition to setting attributes for genomes and links in the input files, users could also add or change attribute values and placement for a genome (e.g. label for genome, label color, label position, showing labels and genomic coordinates or not), or links between two genomes under the flag defined in NGenomeSyn (we defined a flag param of “SetParaFor” for distinguishing setting block, e.g. SetParaFor=global, GenomeALL, Genome1, Link1) in the configuration (Fig. 1a). For a particular layout of genomes, NGenomeSyn provides parameters to control position (MoveToX, MoveToY), rotation (RotateChr), and scaling (ZoomChr) for each genome. For example, we aligned genomes of two rice accessions (“9311” and “ZH11”) against the reference rice genome (“IRGSP”), respectively and adjusted major parameters for scaling, position, and rotation of syntenic regions in a given region of interest (“IRGSP”: Chr6:12914310:18879240), yielding a triangular layout or other layout that clearly showed a structural variation in some of these rice accessions compared to the reference (Fig. 1b and Supplementary Fig. S1). To fit complex layouts of rectangular chromosomes, we designed five styles for links (straight line or Bezier curve) and defined two parameters (StyleUpDown for link start and end at up or down of rectangular sequences in two genomes; HeightRatio for height ratio between two genomes relative to the default value) to control link styles (e.g. StyleUpDown=DownDown of links between “9311” and “ZH1” in Fig. 1d, details were shown in Supplementary Table S1). A more complex layout across seven rice genomes with a similar style presented in Zheng *et al.* (2022) could be easily drawn using NGenomeSyn (Fig. 1c). Besides, we also provided a parameter (e.g. ZoomRegion=Chr6:12914310:18879240 in Fig. 1d) for zooming in on special regions provided by users to explore or show local synteny or genomic variation. For showing gene structures (UTR, CDS) of special regions, we provided a parameter (SpeRegionWidthRatio) for setting ratio relative to the rectangular sequences (Fig. 1e).

NGenomeSyn is originally designed for visualization of synteny on chromosome-level or zooming in on special regions of interest defined by users across any number of genomes. In practice, we recommend users to provide no more than 20 genomes and perform preprocessing (e.g. filtering or trimming fragment assemblies or small syntenic blocks) before using NGenomeSyn. We tested NGenomeSyn using seven rice genomes (Goff *et al.*, 2002; Qin *et al.*, 2021) (genome size: ∼400 Mbp) and two yeast genomes (∼12 Mbp, Genome assembles: GCA_000146045.2 and GCA_000977955.2). NGenomeSyn finished plots within 1 min and used <0.1 GB of RAM for all the tests. Detailed usages and examples have been documented along with the program deposited in the GitHub website.

## 3 Discussion and conclusion

Compared with other visualization tools of synteny on multiple genomes (>3, Jcvi, SynVisio, GENESPACE, plotsr), NGenomeSyn shows a similar flexibility in genome layout as gene-anchored based tools (Jcvi and SynVisio), but showed a higher customization that allows users to set attributes (e.g. color for stroke and fill; ticks and labels for the genomic coordinate) for a specific sequence in a genome (Supplementary Table S2). NGenomeSyn also allows users to define width and color to distinguish different features (UTR, CDS) which is useful in displaying local synteny, genomic variation, and other genomic elements (e.g. TEs) in zooming in on a special region of interest. More importantly, NGenomeSyn adopts more general and simple input formats, making it easy to draw any type of relationships among multiple genomes, e.g. synteny or co-expression as in Fig. 5b of Xu *et al.* (2022).

The NGenomeSyn has already been applied in visualizing genomic or non-genomic relationships in several research studies (Guan *et al.*, 2022; Hou *et al.*, 2022; Xu *et al.*, 2022; Yin *et al.*, 2023). Given its high flexibility and customization, we believe that NGenomeSyn visualization will help researchers to efficiently explore their data and draw publication-quality figures, specifically for those users without advanced computer skills.

## Supplementary Material

btad121_Supplementary_Data
