# Supplementary material for: NGenomeSyn: an easy-to-use and flexible tool for publication-ready visualization of syntenic relationships across multiple genomes
Source: Bioinformatics. 2023 Mar 8;39(3):btad121. doi: 10.1093/bioinformatics/btad121 (PMC10027429; doi:10.1093/bioinformatics/btad121)
Supplement: btad121_Supplementary_Data [file btad121_supplementary_data.docx]

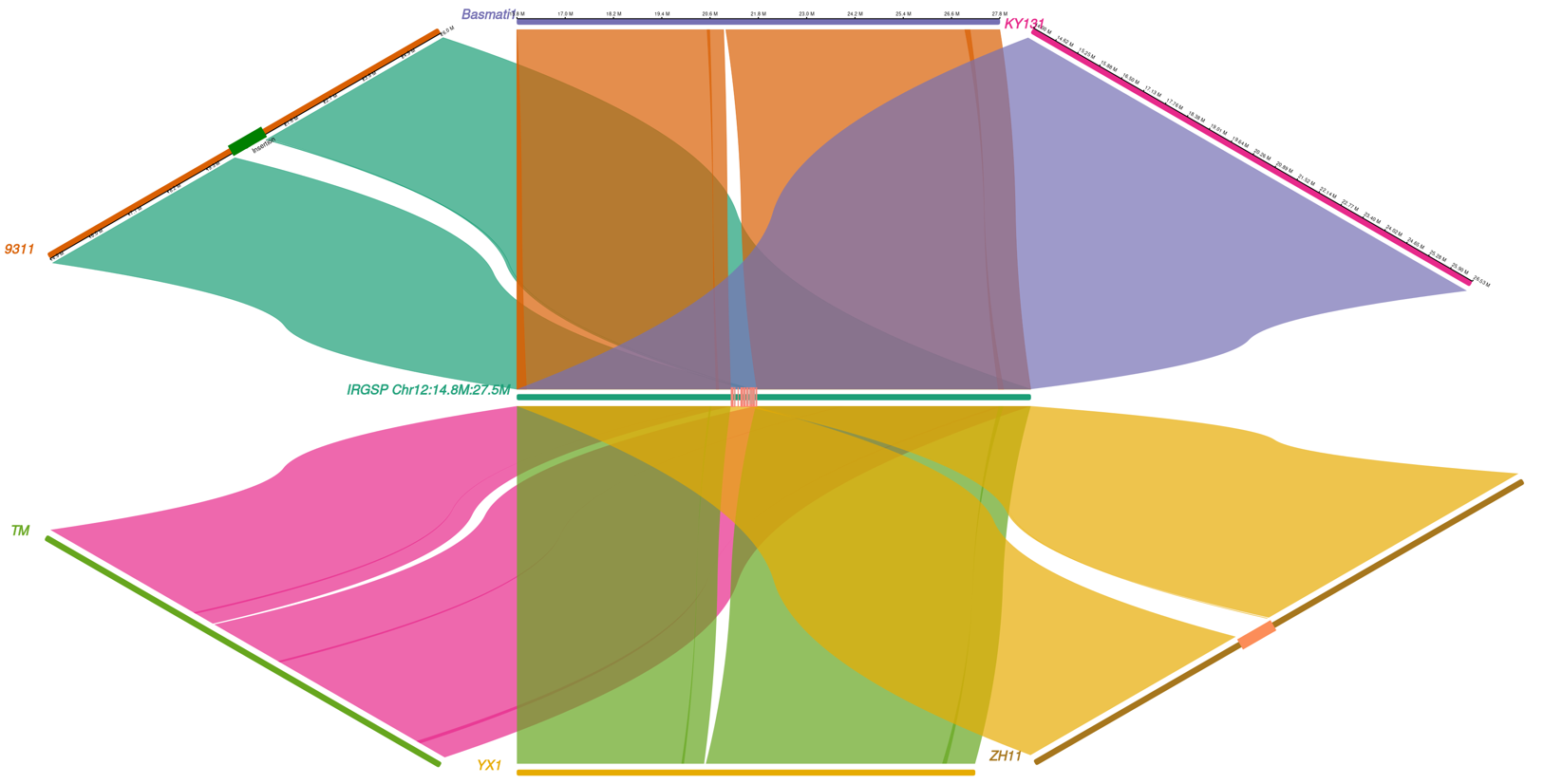


**Supplementary Figure 1. NGenomeSyn showed a segment insertion in two rice accessions (9311 and ZH11) compared to the reference rice genome (IRGSP) in the region of “Chr12:14.8M:27.5M”.**

**Supplementary Table 1. Illustration of five link styles designed in NGenomeSyn.**

| **StyleUpDown** | **Example Figure** |
| --- | --- |
| StyleUpDown=UpDown; HeightRatio=1.0  (**default**) | 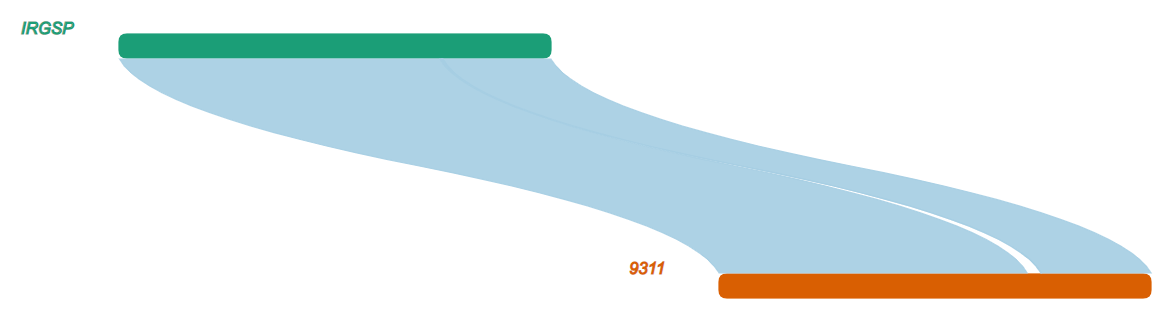 |
| StyleUpDown=UpUp ;HeightRatio=1.5 | 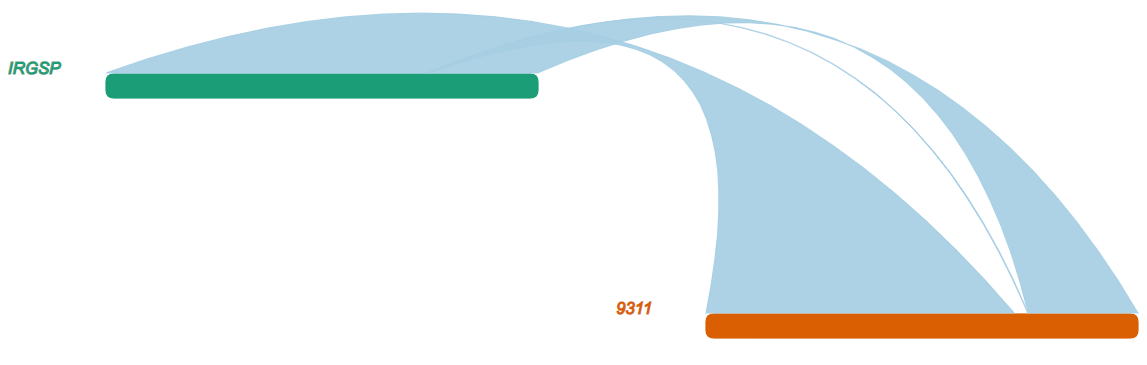 |
| StyleUpDown=DownDown; HeightRatio=1.5 | 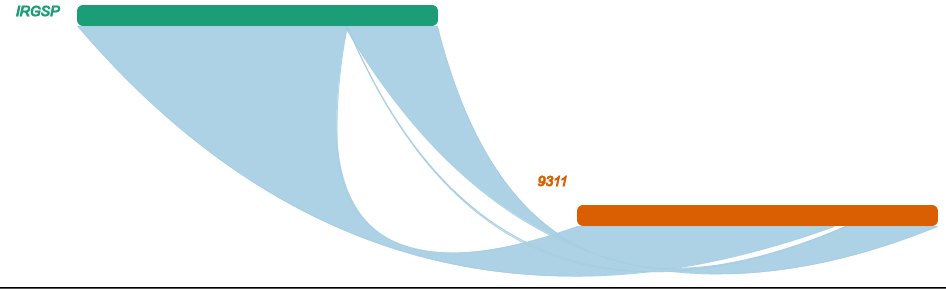 |
| StyleUpDown=DownUp; HeightRatio=1.0 | 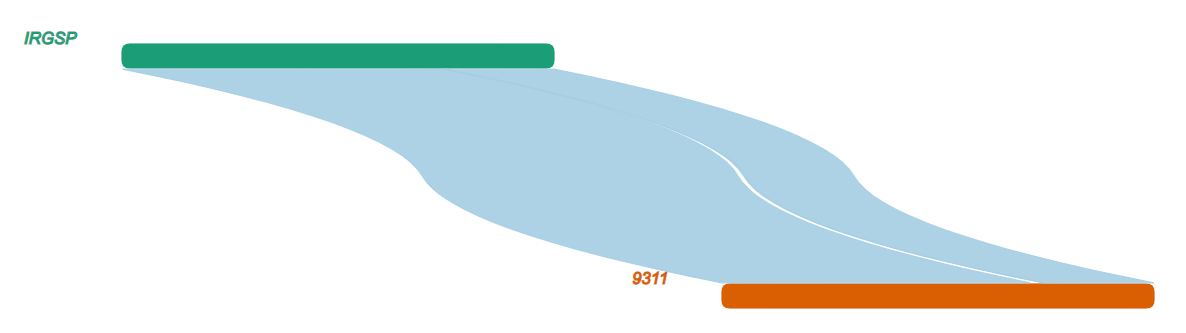 |
| StyleUpDown=line | 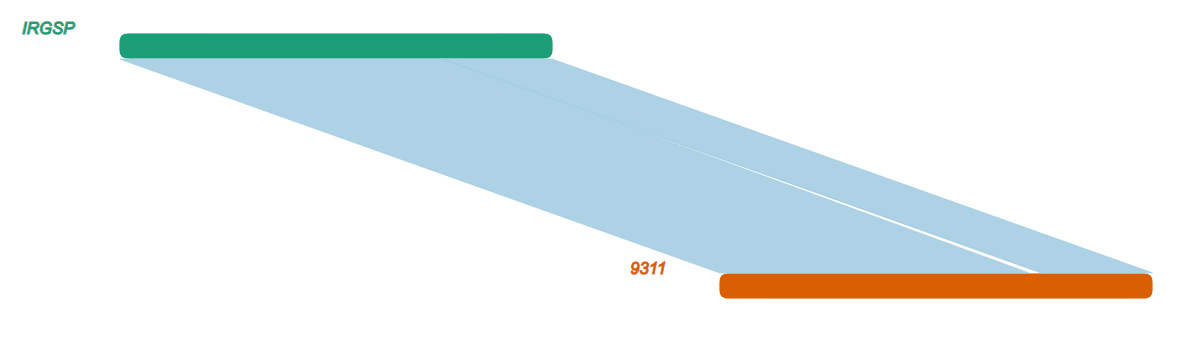 |

|  | NGenomeSyn | Jcvi | SynVisio | plotsr | Genespace | GenomeSyn |
| --- | --- | --- | --- | --- | --- | --- |
| **Language** | Perl | Python | Javascript | Python | R | Perl/Python |
| **Genomes** |  |  |  |  |  |  |
| number (2 and ≥3) | **√** | **√** | **√** | **√** | √ | 2,3 |
| attributes (color,fill,*etc*) | **√** | **√** | **√** | **√** | **√** | **√** |
| **Layout** |  |  |  |  |  |  |
| rotation | **√** | **√** | **√** | **×** | **×** | **×** |
| ZoomChr(scale) | **√** | **√** | **√** | **×** | **×** | **×** |
| movement | **√** | **√** | **√** | **×** | **×** | **×** |
| local region (ZoomRegion) | **√** | **√** | **√** | **×** | **√** | **×** |
| ticks&labels | **√** | **×** | **√** | **√** | **×** | **√** |
| **Special regions** |  |  |  |  |  |  |
| TE, SV, *etc* | **√** | **×** | **×** | **√** | **×** | **√** |
| gene | **√** | **√** | **×** | √ | **×** | **×** |
| gene structure (CDS, UTR ) | **√** | **×** | **×** | **×** | **×** | **×** |
| attributes (color,fill,etc) | **√** | **√** | **-** | √ | **×** | **√** |
| **Links** |  |  |  |  |  |  |
| link file from gene- or genome-based | both | gene | gene | genome | gene | genome |
| **Link styles** |  |  |  |  |  |  |
| linear Bezier curve | **√** | **√** | **√** | **×** | **×** | **×** |
| quadratic Bezier curve | **√** | **√** | **√** | **√** | **√** | **√** |
| straight line | **√** | **√** | **√** | **×** | **×** | **×** |
| attributes (color, fill, etc) | **√** | **√** | **√** | **√** | **√** | **√** |

**Supplementary Table 2. Comparison of NGenomeSyn with other synteny visualization tools.**
